# Effect of Accelerators on the Workability, Strength, and Microstructure of Ultra-High-Performance Concrete

**DOI:** 10.3390/ma15010159

**Published:** 2021-12-26

**Authors:** Yonghua Su, Biao Luo, Zhengdong Luo, He Huang, Jianbao Li, Dehui Wang

**Affiliations:** 1 College of Civil Engineering, Hunan University, Changsha 410082, China; suyonghua1965@163.com; 2 College of Civil Engineering and Mechanics, Xiangtan University, Xiangtan 411105, China; 3 Yueyang City Roads and Bridge Construction Corporation, Yueyang 414002, China; Huanghe6668881@163.com (H.H.); Lijianbao1971@163.com (J.L.); 4 College of Civil Engineering, Fuzhou University, Fuzhou 350116, China; dhwang@fzu.edu.cn

**Keywords:** ultra-high-performance concrete, accelerators, workability, strength, microstructure, hydration products

## Abstract

The preparation of ultra-high-performance concrete (UHPC) with both high-early-strength and good workability contributes to further promotion of its development and application. This study investigated the effects of different accelerators (SM, alkaline powder accelerator; SF, alkaline powder accelerator containing fluorine; and AF, alkali-free liquid accelerator containing fluorine) on the workability and strength properties of UHPC. The microstructure of UHPC was also characterized by using XRD and SEM. Several dosage levels of accelerators (2%, 4%, 6%, and 8% by mass) were selected. The results indicate that the setting time and fluidity of UHPC are gradually decreased with an increase in accelerators dosage. Compared with fluorine-containing SF/AF, fluorine-free SM evidently facilitates UHPC early strength gain speed. However, the fluorine-containing accelerators have a higher 28 d strength ratio, especially AF. The maximum compressive and flexural strength ratios are obtained at a dosage of 6%, which are 95.5% and 98.3%, respectively. XRD and SEM tests further reveal the effect of different accelerators on the macroscopic properties of UHPC from the micro level.

## 1. Introduction

Ultra-high-performance concrete (UHPC) is a cement-based composite material mixed with different active powders and fiber materials [1]. Due to the close packing of various material components and the bridging effect of fibers, UHPC has high compressive strength, good bending toughness, and excellent durability, and has excellent properties such as tensile strain hardening [2,3,4,5]. It is especially suitable for the construction of super long-span and super high-rise structures, as well as the reinforcement of existing concrete structures [5,6,7,8,9,10]. However, many applications, such as shoring, tunneling, rapid repair, and cementing of oil and gas wells require UHPC to have the ability of rapidly setting and hardening.

Relevant studies have found that curing methods such as high-temperature steam curing can accelerate the early hydration process of UHPC, thereby improving the early age mechanical properties of UHPC [11,12,13]. However, on the one hand, the energy consumption of steam curing reaches more than 80% of the total energy consumption of UHPC production, resulting in a huge waste of energy. On the other hand, high-temperature curing easily leads to the deterioration of the microstructure of UHPC in the later stage of hydration. Therefore, it is urgent to prepare UHPC cured at room temperature with both high-early-strength and good workability to promote its innovative and engineering application.

In addition to complex curing methods, accelerators are often used to promote the solidification and early strength development of concrete materials [14]. According to different alkali content, the commonly used accelerators can be divided into alkaline accelerators [15,16] and alkali-free accelerators [17,18]. Renan et al. [17,19] showed that the addition of sodium metaaluminate (NaAlO_2_) would significantly increase the rate of hydration heat release during the induction period, but reduce the rate of heat release after the acceleration period. Meanwhile, the gypsum in the cement was quickly consumed to generate AFm and C-A-H products, but this hindered the dissolution and hydration of C_3_S, which is not conducive to the later strength development of cement-based materials. Han et al. [16] confirmed that although NaAlO_2_ promoted the formation of AFm and improved the early strength of concrete, its alkaline hydrolysate increased the internal pores of the concrete, resulting in a decrease in the later strength. Wang et al. [14,17,18,19] found that aluminum sulfate (Al_2_(SO_4_)_3_), the main component of the alkali-free accelerator, promoted the formation of a large number of acicular ettringite crystals and lapped on the surface of cement particles, which promotes the rapid setting of cement paste. Regardless of whether alkaline or alkali-free accelerators are used, the early setting and hardening rate of concrete is increased in most cases. However, because of the complex material composition of concrete, certain accelerators have poor compatibility with cement, mineral admixtures, and water-reducing admixtures in an actual application. Not only does it not have the effect of early age setting and hardening, but it causes unstable setting time, slow early strength development, and serious late strength loss [20]. In order to improve adaptability, fluorine-containing accelerators based on hydrofluoric acid or fluorine-containing compounds have appeared on the market. Based on the characteristics of fluorine-containing accelerators, the previous literature mainly focused on their influence on the early hydration of cement paste and concrete properties [17]. However, there are few reports on the effects of fluorine-containing accelerators on the properties of UHPC with composite cementitious material systems.

Focusing on this, this study concentrates on the workability and strength properties of UHPC mixed with different accelerators, as well as the composition and microscopic morphology of the early hydration products.

## 2. Materials and Test Program

### 2.1. Materials

P·O 42.5 cement (C), class F fly ash (FA), and silica fume (SF) were used as cementitious materials. Their chemical compositions are summarized in Table 1. Quartz powder (QP, the SiO_2_ content is more than 99.2%) with an average particle size of 50.5 μm was used. The maximum diameter of quartz sand (QS, the SiO_2_ content is more than 99.2%) as aggregate was 2.0 mm. The specific technical indicators of copper-plated steel fiber are shown in Table 2. A powder polycarboxylate-based superplasticizer (PS, the water reducing rate is more than 35%) was used to avoid the increase in water.

To investigate the compatibility and reactivity of different accelerators with UHPC, three types of accelerators were used: (1) alkaline powder accelerator (SM, which is composed of 22.7% Na_2_O, 16.8% Al_2_O_3,_ and 60.5% solid alcohol amine); (2) alkaline powder accelerator containing fluorine (SF, which is composed of Na_2_O, Al_2_O_3_, NaHF_2_, and solid alcohol amine, and their proportions are 26.5%, 14.0%, 2.3%, and 57.2%, respectively); (3) alkali-free liquid accelerator containing fluorine (AF, which is composed of 38.2% Al_2_(SO_4_)_3_, 2.5% HF, and 7.8% alcohol amine). Table 3 shows the technical indicators of three accelerators.

### 2.2. Mixture Proportions

To achieve high strength, a low water–binder ratio (*w*/*b*) of 0.18 was used for all mixtures. The details of the designed mixture proportions are summarized in Table 4 (to ensure a constant *w*/*b*, the design proportions have deducted the moisture content of the liquid accelerator). All the specimens were appointed by both letters and numbers. The letter indicated the accelerator types, and the number indicated the mass fraction of the cementitious materials occupied by the accelerators. The first was the plain mixture without accelerators, which is recorded as P-0. In other mixtures, the letters SM, SF, and AF indicated three different accelerators. The numbers 3 and 6 indicated that the accelerator dosage was 3% and 6%, respectively. For example, SM-3 represents a mixture with 3% SM.

### 2.3. Specimen Fabrication

**Figure 1:**
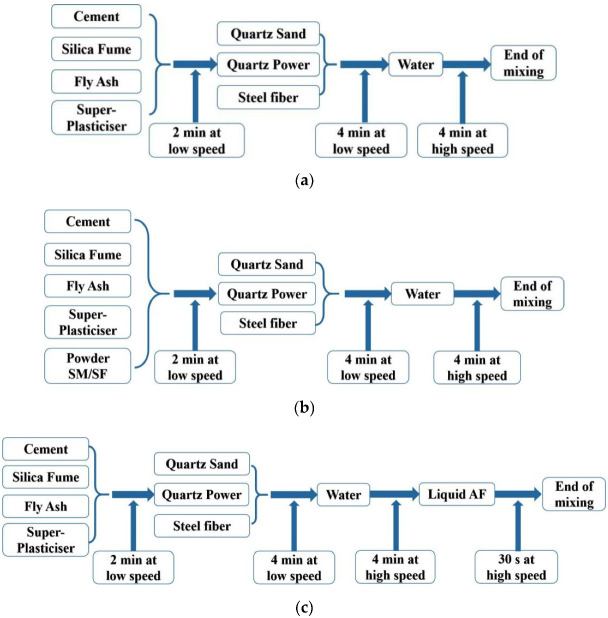
Mixing procedure of UHPC: (**a**) without accelerators; (**b**) with powder SM/SF; (**c**) with liquid AF (low speed: 140 rpm, high speed: 280 rpm).

To achieve uniform UHPC mixtures, the raw materials were sequentially added to the 15 L mixer for mixing according to the preparation procedures shown in Figure 1. The volume of the mixture for each stirring was approximately 10.5 L.

Fresh UHPC mixtures were poured into plastic molds and vibrated continuously for 1.5 min at a frequency of 2200 times/min using a vibrating table. After casting, the specimens with molds were naturally cured for 24 h. Finally, the demolded specimens were transferred to the standard environment with a humidity of 93–97% and a room temperature of 20 °C for curing until required age.

### 2.4. Testing Methods

#### 2.4.1. Setting Time

The setting time of the designed UHPC paste was measured using a Vicat meter (Wuxi Zhongke Building Material Instrument Co., Ltd., Wuxi, China) [21].

#### 2.4.2. Fluidity

The workability of fresh UHPC mixture was evaluated by measuring the fluidity value [21]. Accelerators had the effect of promoting the setting and hardening of concrete. To make each group of data true, reliable, and comparable, the fluidity value tests were supposed to be performed immediately after the UHPC mixtures were stirred. The diameter of the spread UHPC pastes was measured along two perpendicular directions and the calculated average diameter was recorded as the fluidity value.

#### 2.4.3. Mechanical Properties

The compressive strength of 10 cm × 10 cm × 10 cm cube specimen was determined using a TYA-2000A type pressure testing machine (Wuxi Xinluda Instrument Equipment Co., Ltd., Wuxi, China) with the maximum load of 2000 kN. The flexural strength test was performed using an electro-hydraulic servo universal testing machine (Wuxi Xinluda Instrument Equipment Co., Ltd., Wuxi, China) with a measuring range of 1000 kN, and the size of specimens tested was 40 cm × 10 cm × 10 cm. The strength obtained was the average of three repeated tests [21].

#### 2.4.4. X-ray Diffraction (XRD)

The XRD test was used to analyze the early hydration mechanism of UHPC in the presence of different accelerators. The selected UHPC paste samples excluded quartz powder, quartz sand, and steel fibers. The test samples ground into powders (<40 μm) were soaked in ethanol liquid for 2 d to prevent hydration, and then dried in a vacuum oven at a temperature of 45 °C until the weight remained unchanged. The powders were examined by a PANalytical x’pert Pro diffractometer (PANalytical B.V., Almelo, The Netherlands) and the scanning angle was 5° to 45° with a speed of 1° per 6 s.

#### 2.4.5. Scanning Electron Microscope (SEM)

The Zeiss GeminiSEM 300 microscope (Carl Zeiss AG, Oberkochen, Germany) was used to analyze the morphology changes of the hydration products of UHPC pastes at 1 d and 3 d of hydration. In the SEM test, the method of hydration termination and drying treatment of the samples (about 10 mm in diameter and 8 mm in thickness) was consistent with the pretreatment method of XRD test. Before testing, the samples were sprayed with gold.

## 3. Results and Discussion

### 3.1. Setting Time

**Figure 2:**
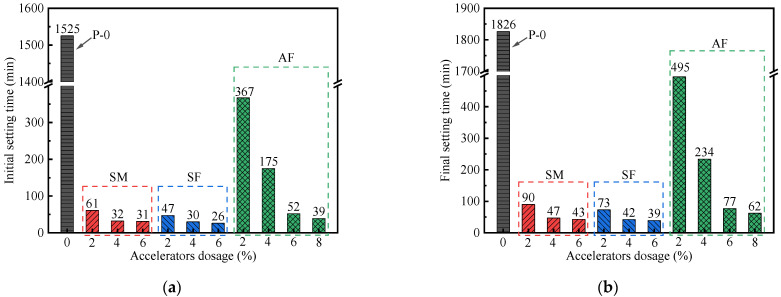
Setting time of UHPC pastes: (**a**) initial setting time; (**b**) final setting time.

Figure 2 represents the variation laws of setting time of different UHPC with SM/SF/AF dosage range. It could be seen that the incorporation of accelerators significantly reduces the setting time of these UHPC, and the acceleration effect of SF is the most obvious. In Figure 2a,b, when the SM dosage increases from 2% to 6%, the setting times of UHPC pastes gradually become stable. As for UHPC pastes mixed with SF, their initial and final setting times are less than 47 min and 73 min, respectively. It shows that the setting times of UHPC pastes mixed with the same dosage of fluorine-containing SF are shorter than that of SM doped. In other words, to achieve the same acceleration effect, the lower dosage of fluorine-containing accelerators is required. This may be due to the fact that F^−^ ions (fluoride ions) are beneficial to dissolve C_3_S and accelerate the C_3_A hydration [22,23]. As shown in Figure 2a,b, although AF can also reduce the setting times of UHPC, the overall effect is weaker than SM and SF. When the AF dosage increases from 2% to 6%, the setting times of UHPC pastes are continuously shortened, but when the AF dosage continues to increase, the setting times of UHPC pastes gradually become stable.

### 3.2. Fluidity

**Figure 3:**
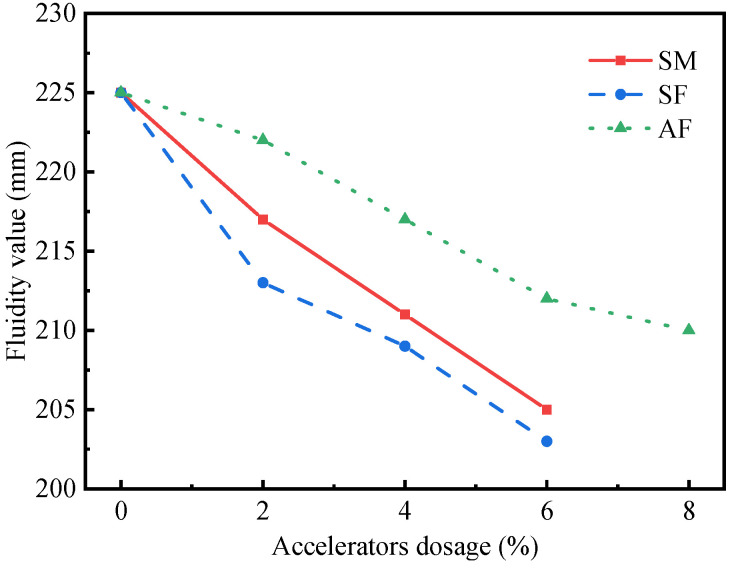
Fluidity value of UHPC pastes.

The test results of UHPC paste fluidity test are shown in Figure 3. It can be found that the fluidity of UHPC paste presents a trend similar to the setting time. Specifically, the UHPC pastes’ fluidity are gradually decreased with increasing SM, SF, and AF dosages. In the presence of 2% SM, the UHPC paste fluidity is 217 mm. When the SM dosages are 4% and 6%, the paste fluidity are 211 mm and 205 mm, respectively. This may be mainly due to the presence of accelerators that promote the rapid production of hydration products of UHPC paste during the initial hydration process. Therefore, a lot of free water is fixed in the form of crystal water, which is equivalent to reducing the initial mixing water content of UHPC pastes. This hinders the flow of the paste, resulting in a decrease in fluidity. In addition, the fluidity value of UHPC pastes mixed with SM are slightly higher than that of mixed SF, but lower than that of mixed AF. For instance, when the accelerators dosage is 2%, the fluidity of UHPC pastes mixed with AF, SM, and SF are 222 mm, 217 mm, and 213 mm, respectively. It should be pointed out that the accelerators dosage should be strictly controlled in practical application to ensure that the high-early-strength UHPC has good workability.

### 3.3. Compressive Strength

**Figure 4:**
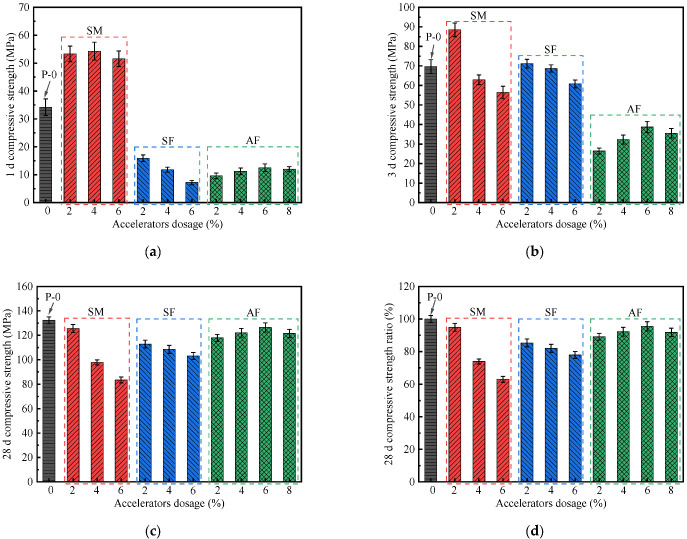
Effect of accelerators on the compressive strength of UHPC: (**a**) 1 d, (**b**) 3 d, and (**c**) 28 d compressive strength; (**d**) 28 d compressive strength ratio.

The early and late strength of concrete mixed with accelerators can accurately reflect its application performance [22]. Based on this, the 1 d, 3 d, and 28 d compressive strengths of UHPC in the presence of different accelerators were measured. The specific test results are shown in Figure 4.

As shown in Figure 4a, the addition of SM significantly improves the early compressive strength of UHPC, and different dosages will lead to different improvement effects. When the SM dosage increases from 2% to 6%, the 1 d compressive strength increases by 50.9–58.8% compared with P-0. SM-4 obtains the largest 1 d compressive strength of 54.3 MPa. Previous studies have shown that hydration degree is one of the important factors affecting the early strength of cementitious material systems [24]. Therefore, the increase in early strength is due to the SM accelerating the hydration process of UHPC, and its compressive strength is positively correlated with its hydration degree. On the contrary, the rapidly formed dense hydration product has a coating inhibitory effect on the unhydrated cementitious material particles, which hinders the further hydration of UHPC [17]. At 3 d of hydration (Figure 4b), as the SM dosage increases, the 3 d compressive strength of UHPC continuity decreases. It is clear that 2% SM can improve 3 d compressive strength, while adding a higher dosage of SM will have a significant negative effect on the strength development. Specifically, the 3 d compressive strength of SM-2 is 88.5 MPa, which is approximately 27.0% higher than P-0. In addition, compared with P-0, the 3 d compressive strengths of SM-4 and SM-4 are decreased by 9.8% and 19.1%, respectively. At 28 d (Figure 4c,d), the compressive strength of UHPC mixed with SM is in the range of 97.8–125.6 MPa. It is equivalent to 73.9–94.9% of the compressive strength of P-0. The 28 d compressive strength ratio is found the highest for the SM-2 (about 94.9%). It can be seen that the strength ratio of UHPC decreases with the increase in SM dosage. This may be due to the addition of SM, which limits the filling and refinement of the micro-pores of UHPC matrix in the later stage of hydration, thereby increasing the porosity of the matrix structure [16,25,26].

The results in Figure 4a show that the incorporation of fluorine-containing SF significantly decreases the 1 d compressive strength of UHPC. An amount of 6% SF addition results in a sharp reduction (78.9%) in compressive strength, whereas 2% SF addition also results in a 53.5% strength loss at the end of the first day. This is due to the alkali content of SF being relatively high, and the large amount of strong alkaline hydroxide released after dissolution makes the hydration product loose and porous [27]. Meanwhile, hydroxide combines with Ca^2+^ ions to form insoluble calcium salt or calcium hydroxide, and the adsorption or intercalation effect of CaF_2_, which adversely affect the early strength development of UHPC [22]. Figure 4b shows that the compressive strength of UHPC mixed with SF increases significantly with age. Furthermore, the 3 d compressive strength of SF-2 is 2.2% higher than that of P-0. Except for SF-2, the 28 d compressive strength ratios of all other UHPC mixed with SF are higher than that of UHPC mixed with SM, and their compressive strength ratios are above 77.9%.

As for UHPC with AF, it can be seen in Figure 4a,b that the addition of AF is not conducive to the early strength development of UHPC, so that the compressive strength remains at a low level in the early stage of hydration (within 3 days). When the AF dosage is increased from 2% to 8%, the 1 d compressive strength of UHPC is between 9.6 MPa and 12.5 MPa, and the 3 d compressive strength is between 26.4 MPa and 38.7 MPa. The reasons for the slow development of strength may be as follows: (1) the adsorption behavior of comb-shaped polycarboxylic molecules retards the acceleration effect of liquid AF on UHPC [28]; (2) the F^−^ ion in the AF reacts with Ca^2+^ ion in the UHPC pastes to produce poorly soluble CaF_2_ products, and CaF_2_ products have adsorption or intercalation effect on C-S-H gel [29], which evidently delays the hydration rate of UHPC. From 3 d to 28 d, a manifest increase in compressive strength can be observed. This may be due to the accelerated hydration reaction of powder particles. In Figure 4c, the 28 d compressive strength ratios of all UHPC with AF are above 89.1%. It should be emphasized that the 28 d compressive strength ratio of UHPC with 6% AF is the highest, reaching 95.5%.

### 3.4. Flexural Strength

**Figure 5:**
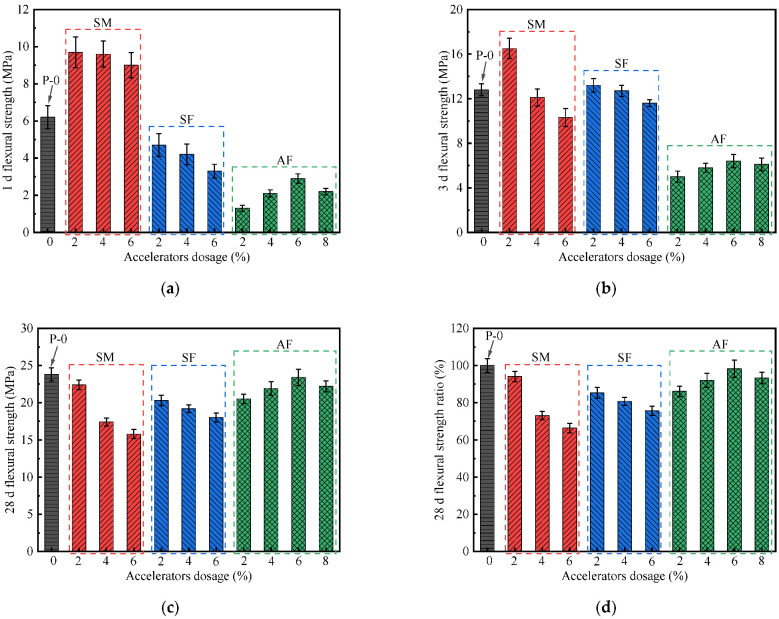
Effect of accelerators on the flexural strength of UHPC: (**a**) 1 d, (**b**) 3 d, and (**c**) 28 d flexural strength; (**d**) 28 d flexural strength ratio.

The influence of accelerators on flexural strength of UHPC is presented in Figure 5. It shows that the development law of flexural strength and compressive strength is similar, which indicates that there is an explicit effect of the matrix strength on the fiber–matrix bond [30,31,32]. From these figures, the 1 d flexural strength after 2% SM adding is obviously higher than that of P-0, with the maximum increment of up to 56.5%. The 3 d flexural strength of SM-2 is 16.5 MPa, which corresponds to an increase of 28.9%. The flexural strength increment starts to decrease when SM dosage increases from 2% to 6%. The 1 d flexural strength is increased by 54.8% by incorporation of 4% SM, while this increment falls to 45.2% when 6% SM is incorporated. For UHPC with SM cured for 3 d, a 5.5–19.5% decrease in flexural strength happens when the SM dosage is higher than 4%. The higher the dosage is, the more serious the flexural strength decrease is. This phenomenon is similar to the development law of UHPC compressive strength. At 28 d, compared with P-0, the flexural strength ratios of SM-2, SM-4, and SM-6 are 94.1%, 73.1%, and 66.4%, respectively. It is shown that the 28 d flexural strength of P-0 is higher than that of UHPC mixed with SM. This is because the strength of the UHPC matrix without accelerators has been fully developed, and the toughening effect of steel fibers has been further enhanced.

From Figure 5, it shows that the order of 1 d flexural strength of UHPC is as follows: SM > P-0 > SF > AF. In the presence of different SF dosages, the 1d flexural strength is 48.5%, 43.8%, and 36.7% of UHPC mixed with SM, respectively. When AF dosage is 2–8%, the 1 d flexural strength of UHPC is between 1.3 MPa and 2.9 MPa, which is much lower than P-0. This indicates that F^−^ ion has a strong weakening effect on the early hardening strength development of UHPC. It is obvious from Figure 5c,d that all fluorine-containing accelerators have a higher 28 d flexural strength ratio, especially AF. The maximum strength ratio is obtained at a dosage of 6%, which is 98.3%.

### 3.5. XRD Pattern

**Figure 6:**
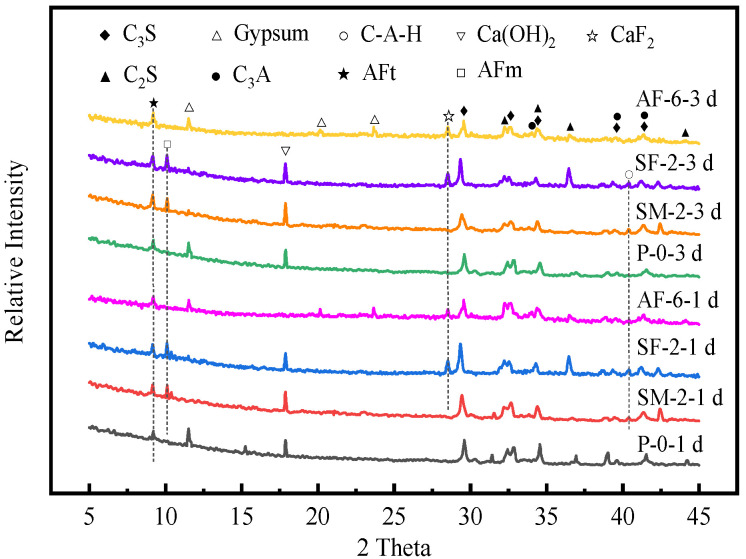
XRD pattern of hydration products of UHPC.

The type of hydration product is closely related to the main components of accelerators. The above-mentioned studies have shown that 2% SM, 2% SF, and 6% AF have similar acceleration effects on UHPC. In order to further analyze the influence of accelerator type on the phase composition of UHPC hydration products, XRD analysis was performed on their hydration products at 1 d and 3 d. The obtained XRD patterns are shown in Figure 6.

Figure 6 illustrates the XRD test results of P-0. Along with the hydration process, the gypsum in UHPC is gradually consumed, accompanied by the production of Ca(OH)_2_. The AFt diffraction peak is identified at 1 d after UHPC is stirred with water. With the further hydration of UHPC, the diffraction pattern of the P-0 at 3 d of hydration is roughly similar to that at 1 d. The difference is that the peak intensity of AFt gradually increases, while the peak intensities of C_3_S, C_2_S, C_3_A, and gypsum are weaker than at 1 d.

As shown in Figure 6, due to the lack of _${\mathrm{SO}}_{4}^{2-}$_ in SM, gypsum is consumed significantly after the use of SM. Meanwhile, AFm is identified at 1 d and the peak intensity of AFm gradually increases with time, which suggests that incorporation of SM expedites the formation of AFm. In addition, the diffraction peak of C-A-H is found. C-A-H and AFm products are more thermally stable and difficult to dissolve than AFt. They cover the surface of the powder particles and hinder subsequent hydration [16,17]. This is the main reason why the strength development of UHPC containing SM slows down in the later stage.

In Figure 6, when SF is used, a large amount of AFm and C-A-H are generated after 1 d of reaction, and the gypsum peak intensity is weaker than P-0 and the peak intensity of AFm gradually increases with age. The reason is that after adding SF into UHPC, the ratio of sulfate to aluminate decreases drastically and AFm is formed rapidly. Meanwhile, an apparent CaF_2_ diffraction peak is found in the spectrum at 1 d. Combined with the above-mentioned mechanical performance test results, it is proved that F^−^ participates in the early hydration process of UHPC. The reasons for its influence on the early strength of UHPC are, on the one hand, the generated CaF_2_ product has an encapsulation and adsorption effect on the C_3_S or C-S-H gel and increases the steric hindrance of the migration of liquid ions in the paste to the surface of the powder particles, thereby delaying the subsequent hydration process of UHPC. On the other hand, F^−^ and Al^3+^ form a stable aluminum fluoride complex structure, leading to a slow formation of [Al(OH)_4_]^−^, and thus delaying the nucleation process of ettringite.

As for AS, its main components are Al^3+^, _${\mathrm{SO}}_{4}^{2-}$_, and H^+^, which provides a large amount of sulfate for UHPC hydration. After adding AS, Ca(OH)_2_ will be consumed during the hydration process to form AFt [17]. Additionally, similar to the XRD spectrum of SF-2, AF-6 also has a CaF_2_ peak. Combined with the decrease in the early strength of UHPC containing AF, it further proves that F^−^ has a weakening effect on the early strength development of UHPC.

### 3.6. SEM Examination

**Figure 7:**
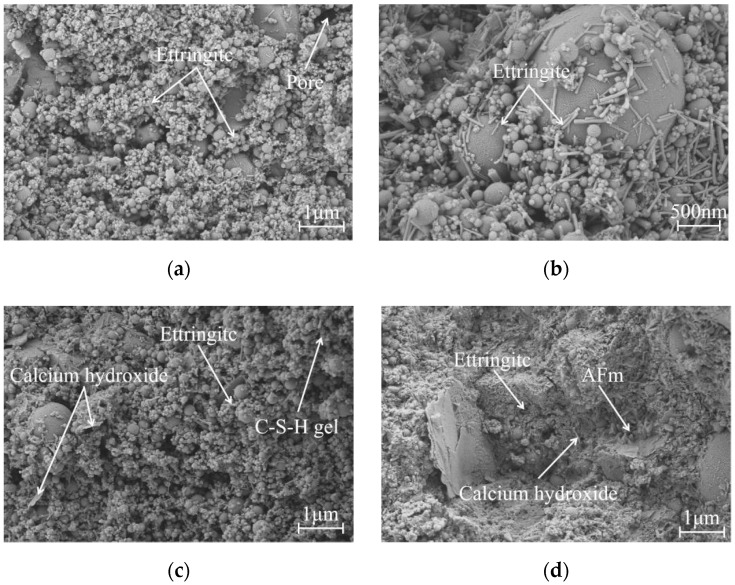

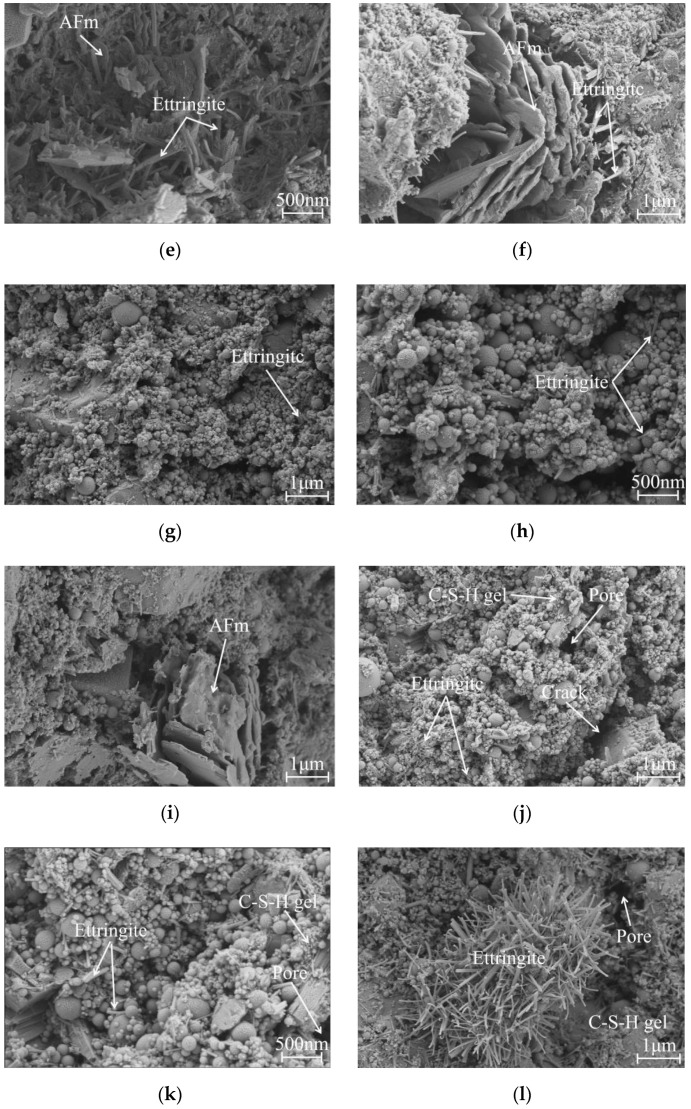
Morphology of the hydration products of UHPC: (**a**,**b**) P-0, 1 d; (**c**) P-0, 3 d; (**d**,**e**) SM-2, 1 d; (**f**) SM-2, 3 d; (**g**,**h**) SF-2, 1 d; (**i**) SF-2, 3 d; (**j**,**k**) AF-6, 1 d; and (**l**) AF-6, 3 d.

To further reveal the effect of different accelerators on the macroscopic properties of UHPC from the microstructure level, SEM test was used to characterize the microstructure evolution of UHPC at 1 d and 3 d of hydration. The SEM images of UHPC are shown in Figure 7, and the main characteristic hydration products are marked. Comparing the morphology of UHPC under the same hydration age, it is found that the accelerators type significantly affects the early microstructure development of UHPC hardened paste. The difference in microstructure characterization is a good proof of the aforementioned UHPC mechanical properties test results.

As can be seen from Figure 7a,b, when the P-0 without accelerators is hydrated for 1 d, only a little gel product and acicular ettringite (AFt) crystals are formed on the surface of the cementitious material particles, so that they cannot be completely wrapped. Meanwhile, there is coarse pore known as a harmful pore that can be observed, which affects the compactness of the microstructure [16]. As highlighted in Figure 7c, the compactness of the microstructure of P-0 at 3 d has been improved. There are tabular calcium hydroxide (Ca(OH)_2_) products intersperse in the micropores. Besides, the number of AFt crystals has increased significantly and they overlap each other on the surface of the microstructure.

A different microstructure is observed in SM-2 (Figure 7d–f). At 1 d, irregular tabular hydration products (later identified as AFm) are generated, this is consistent with the result of the XRD pattern. Although AFt crystals are found in small crevices, AFm is still the main hydration product and has become more regular with time. In addition, the SM contains alkaline components, which can stimulate the pozzolanic activity of supplemental cementitious materials (fly ash and silica fume). Therefore, UHPC has a high degree of hydration and basically forms a relatively dense gel system. This also further verifies that the UHPC matrix mixed with SM has a higher early strength. Meanwhile, the increase in the matrix strength contributes to enhance the bonding behavior between the UHPC matrix and the steel fiber, thereby enhancing the bridging effect of the steel fiber. As a result, it is manifested as an increase in flexural strength. It can be seen from Figure 7f that the microshape of AFm has transformed irregular tabular and floccule shapes into rose petals (multiple flake crystals). Meanwhile, the coarse pores are gradually filled with hydration products such as AFt crystals.

After using SF (Figure 7g,h), little micropores are observed in the microscopic morphology of the hydration products at 1 d, which is speculated to be one of the reasons for the early strength reduction of UHPC mixed with SF. This is due to the high alkali content of the SF, and the strong alkaline hydrolyzate produced makes the UHPC hydration products loose and porous [28]. With the progress of the hydration reaction, SF also accelerates the formation of the AFm phase (Figure 7i), which is basically the same as the SM. The generated AFm product is petal-like and filled in the pores of the UHPC microstructure, which improves the compactness of the microstructure.

Compared with others, the addition of AF results in a relatively loose and porous microstructure of UHPC (Figure 7j–l). Although a lot of AFt crystals are rapidly produced within 1 d, most of them are loosely distributed on the surface of the microstructure and the micropores and microcracks are not effectively filled. The defects of microstructure support the reason for the lower early strength of UHPC mixed with AF.

## 4. Conclusions

(1) The setting time and fluidity of UHPC are gradually decreased with increase in accelerators dosage, and the order of acceleration effect is as follows: SF > SM > AF. From the perspective of setting time, 2% SM, 2% SF, and 6% AF have similar acceleration effects on UHPC. The accelerator promotes the formation of hydration products, resulting in a lot of free water being fixed in the form of crystal water, thereby decreasing the fluidity of the UHPC paste.

(2) The addition of different types and dosages of accelerators causes significant differences in the strength development of UHPC. For SM/SF, the early strength of UHPC generally decreases with the increase in the accelerator dosage. As for AF, its strength first increases and then decreases, and the maximum strength is obtained at 6%. In the case of the same dosage of accelerators, the order of early strength of UHPC from high to low is as follows: SM > P-0 > SF > AF. It shows that F^−^ ion has a weakening effect on the early strength development of UHPC. However, the fluorine-containing accelerators have a higher 28 d strength ratio, especially AF. The maximum compressive and flexural strength ratios are obtained at a dosage of 6%, which are 95.5% and 98.3%, respectively.

(3) XRD and SEM tests further prove that the morphology and phase composition of the hydration products are closely related to the main components of accelerators. After adding SM/SF into UHPC, the ratio of sulfate to aluminate decreases drastically and AFm is formed rapidly. Meanwhile, the CaF_2_ diffraction peaks are observed in both SF and AF, which confirms that F^−^ participates in the early hydration process of UHPC. However, the presence of F^−^ delays the nucleation process of ettringite, and the CaF_2_ product has an encapsulation and adsorption effect on the C_3_S or C-S-H gel, which hinders the effective filling of micropores and microcracks.

## Figures and Tables

**Table 1:** Chemical composition of cementitious materials (wt %).

Materials	SiO_2_	Fe_2_O_3_	CaO	Al_2_O_3_	MgO	K_2_O	Na_2_O	SO_3_
C	21.00	2.90	65.40	5.50	3.30	-	-	2.00
SF	94.20	0.57	0.64	0.30	0.28	0.87	0.16	-
FA	49.20	1.30	3.13	27.80	0.85	-	-	1.21

**Table 2:** Technical indicators of steel fiber.

Length/mm	Equivalent Diameter/mm	Density/(g·cm^−3^)	Tensile Strength/MPa	Elastic Modulus/GPa
13	0.2	7.85	2000	750

**Table 3:** Technical indicators of different accelerators.

Type	Fineness (80 μm Sieve Residue)/%	pH/%	Solid Content/%	Density /(g·cm^−3^)	Cl- /%	Setting Time/min		1 d Strength/MPa	28 d Strength Ratio/%
						Initial	Final		
SM	11.5	-	-	-	0.06	2.5	7.1	8.2	90
SF	10.0	-	-	-	0.04	2.6	8.0	10.5	75
AS	-	3.8	48.5	1.47	0.06	3.2	7.0	7.6	90

**Table 4:** Mixture proportions of the designed UHPC (kg/m^3^).

NO.	C	SF	FA	QS	QP	Steel Fiber	*w*/*b*	PS (wt %)	SM (wt %)	SF (wt %)	AF (wt %)
P-0	783	196	78	861	235	158	0.18	0.5	-	-	-
SM-2	783	196	78	861	235	158	0.18	0.5	2	-	-
SM-4	783	196	78	861	235	158	0.18	0.5	4	-	-
SM-6	783	196	78	861	235	158	0.18	0.5	6	-	-
SF-2	783	196	78	861	235	158	0.18	0.5	-	2	-
SF-4	783	196	78	861	235	158	0.18	0.5	-	4	-
SF-6	783	196	78	861	235	158	0.18	0.5	-	6	-
AF-2	783	196	78	861	235	158	0.18	0.5	-	-	2
AF-4	783	196	78	861	235	158	0.18	0.5	-	-	4
AF-6	783	196	78	861	235	158	0.18	0.5	-	-	6
AF-8	783	196	78	861	235	158	0.18	0.5	-	-	8

## Data Availability

Not applicable.
